# Clinicopathological characteristics of histiocytic sarcoma affecting the central nervous system in dogs

**DOI:** 10.1111/jvim.15673

**Published:** 2020-01-10

**Authors:** Izumi Toyoda, William Vernau, Beverly K. Sturges, Karen M. Vernau, John Rossmeisl, Kurt Zimmerman, Chelsea M. Crowe, Kevin Woolard, Michelle Giuffrida, Robert J. Higgins, Peter J. Dickinson

**Affiliations:** ^1^ Veterinary Medical Teaching Hospital School of Veterinary Medicine, University of California‐Davis Davis California; ^2^ Department of Pathology, Microbiology and Immunology University of California Davis, School of Veterinary Medicine Davis California; ^3^ Department of Surgical and Radiological Sciences University of California Davis, School of Veterinary Medicine Davis California; ^4^ Department of Small Animal Clinical Sciences Virginia‐Maryland College of Veterinary Medicine Blacksburg Virginia

**Keywords:** canine, central nervous system, cerebrospinal fluid, neoplasia

## Abstract

**Background:**

Histiocytic sarcoma affecting the central nervous system (CNS HS) in dogs may present as primary or disseminated disease, often characterized by inflammation. Prognosis is poor, and imaging differentiation from other CNS tumors can be problematic.

**Objective:**

To characterize the clinicopathological inflammatory features, breed predisposition, and survival in dogs with CNS HS.

**Animals:**

One hundred two dogs with HS, 62 dogs with meningioma.

**Methods:**

Retrospective case series. Records were reviewed for results of cerebrospinal fluid (CSF) analysis, CBC, treatment, and outcome data.

**Results:**

Predisposition for CNS HS was seen in Bernese Mountain Dogs, Golden Retrievers, Rottweilers, Corgis, and Shetland Sheepdogs (*P* ≤ .001). Corgis and Shetland Sheepdogs had predominantly primary tumors; Rottweilers had exclusively disseminated tumors. Marked CSF inflammation was characteristic of primary rather than disseminated HS, and neoplastic cells were detected in CSF of 52% of affected dogs. Increased neutrophil to lymphocyte ratios were seen in all groups relative to controls (*P* <.008) but not among tumor subtypes. Definitive versus palliative treatment resulted in improved survival times (*P* < .001), but overall prognosis was poor.

**Conclusions and Clinical Importance:**

Clinicopathological differences between primary and disseminated HS suggest that tumor biological behavior and origin may be different. Corgis and Shetland Sheepdogs are predisposed to primary CNS HS, characterized by inflammatory CSF. High total nucleated cell count and the presence of neoplastic cells support the use of CSF analysis as a valuable diagnostic test. Prognosis for CNS HS is poor, but further evaluation of inflammatory mechanisms may provide novel therapeutic opportunities.

**Abbreviations**
APC: antigen presenting cell
CI: confidence interval
CNS: central nervous system
CSF: cerebrospinal fluid
HS: histiocytic sarcoma
IQR: interquartile range
MST: median survival time
N:L: neutrophil to lymphocyte ratio
RBC: red blood cell
TNCC: total nucleated cell count
TP: total protein concentration

## 1 INTRODUCTION

Histiocytic sarcomas (HS) are tumors that arise typically from interstitial dendritic cells^1^, ^2^ and may occur as localized disease or as disseminated disease affecting multiple organs. Several dog breeds are consistently overrepresented in HS case series, and a familial association has been reported in Bernese Mountain Dogs.^3^, ^4^, ^5^ Defined breed predisposition data for HS are limited, but Bernese Mountain Dog, Golden Retriever, Flat Coat Retriever, Shar Pei, Miniature Schnauzer, Labrador Retriever, and Pembroke Welsh Corgi breeds have been shown to be overrepresented based on defined control populations,^6^, ^7^ and Rottweilers are conspicuously overrepresented in several case series.^1^, ^8^, ^9^, ^10^, ^11^ Histiocytic sarcoma involving the central nervous system (CNS) is relatively uncommon representing 2.2% (n = 2) of primary and 3.4% (n = 7) of secondary intracranial neoplasms diagnosed at necropsy,^12^ and prognosis regardless of treatment is anecdotally poor.^13^, ^14^, ^15^ Intracranial CNS HS commonly are reported as extra‐axial, uniformly contrast‐enhancing masses on magnetic resonance (MR) imaging, and differentiation from more commonly occurring, and generally more benign extra‐axial tumors such as meningioma can be challenging.^13^, ^16^, ^17^

The majority of published data about CNS HS relates to primary, localized CNS HS in small case series,^13^, ^14^, ^15^, ^16^, ^17^, ^18^, ^19^ with limited availability of defined breed predisposition data.^13^ Pembroke Welsh Corgis, Shetland Sheepdogs, Labrador Retrievers, and Golden Retrievers were overrepresented relative to total hospital population in 1 study,^13^ and Corgis also were noted at higher frequencies in other uncontrolled studies.^18^, ^19^ Central nervous system HS appear to differ from HS in most other organs in the severity of inflammatory infiltrate,^2^, ^13^, ^15^, ^18^, ^19^ but it is unclear whether this inflammatory phenotype is common to all CNS HS or if it is restricted to primary localized disease. Markedly increased total nucleated cell counts (TNCC) have been reported in cerebrospinal fluid (CSF) from CNS HS cases.^13^, ^20^, ^21^ This observation likely reflects the inflammatory component of the disease and is in contrast to most other primary CNS tumors in which normal to mildly increased CSF cell counts are typical.^22^, ^23^, ^24^

To further evaluate the inflammatory nature of CNS HS in dogs, a clinicopathological review of a series of primary and disseminated CNS HS cases and meningiomas was done. Markers of inflammation in CSF (TNCC) and peripheral blood (leukocyte neutrophil to lymphocyte [N:L] ratio) were defined in the context of tumor subtype (primary or disseminated CNS HS, meningioma) to determine potential relevance to diagnostic and prognostic criteria.

## 2 MATERIALS AND METHODS

This was a retrospective investigational study conducted at the University of California (UC) Davis School of Veterinary Medicine and Department of Small Animal Clinical Sciences, Virginia Maryland College of Veterinary Medicine. Medical records were reviewed for cases with a primary inclusion criterion of a histopathological diagnosis of HS affecting the CNS. Definitive classification as primary CNS (either as a solitary mass or multifocal disease restricted to the CNS) or disseminated (CNS disease with evidence of other organ system involvement) required full necropsy including the CNS. Cases were classified as brain or spinal cord based on primary neuroanatomical localization at presentation. Additional clinical and clinicopathological data were collected including signalment, clinical history, CSF analysis, CBC, treatment history, and survival.

### 2.1 Pathology

Diagnosis of HS was made by a board‐certified pathologist (R.J.H. or K.W.) based on previously described histopathological and immunophenotypic features.^1^, ^2^

### 2.2 Breed predisposition

Overrepresentation of HS in specific breeds was determined only for dogs diagnosed with CNS HS (n = 91) admitted to the UC Davis Veterinary Medical Teaching Hospital (VMTH) compared to the total UC Davis VMTH reference population (n = 222 814) during the study period (1986‐2018).

### 2.3 CSF analysis

Cerebrospinal fluid analyses were included from cases in which samples were acquired at first presentation, before specific treatment. The CSF collection site was noted when available. Any corticosteroid administration within 1 week of CSF analysis was noted. Total nucleated cell counts, total protein concentration (TP), RBC counts, and differential counts were performed within 30 minutes of collection of CSF as previously described.^23^ Three cases of HS CSF analysis (primary brain cases 14, 30; primary spinal cord case 5) and 56 reference CSF analyses from intracranial meningioma cases were reported previously.^21^, ^23^, ^25^ Cytocentrifuge Wright's or Wright's‐Giemsa stained preparations were retrospectively reviewed by 2 board‐certified clinical pathologists (W.V., K.Z.) for the presence of neoplastic cells and mitotic figures.

### 2.4 Neutrophil to lymphocyte (N:L) ratio

Inclusion criteria for N:L ratios were the same as for CSF analysis. Complete blood counts from cases that had received corticosteroids within 1 week of presentation were excluded from analysis. Only VMTH cases were included, and reference values for N:L ratios were calculated from raw CBC reference interval data from the UC Davis VMTH clinical laboratory.

### 2.5 Treatment and outcome

Treatment groups were defined as definitive if specific treatment modalities including surgical resection, radiation treatment, or chemotherapy (specifically lomustine) were used in any combination, and palliative if anti‐inflammatory (non‐steroidal), corticosteroid, antiepileptic, or other analgesic medications were the only therapeutic interventions. Survival was defined for all cases from time of presentation with neurological signs, and only for cases with survival times >1 day to exclude animals euthanized at the time of imaging or diagnosis.

### 2.6 Statistical analysis

To define breed predisposition, a Fisher's exact test was used to compare the distribution of HS within each breed relative to the entire population of dogs admitted to the UC Davis VMTH during the retrospective period, and the corresponding odds ratios were presented with surrounding 95% confidence intervals (CI).

Continuous variables were assessed for normal distribution using a combined test of skewness and kurtosis. Rates of steroid administration were compared between groups using Fisher's exact test. Cerebrospinal fluid, TP, TNCC, and CBC N:L ratios were compared according to steroid administration status within the overall population and within histiocytic and meningioma subgroups using Wilcoxon rank sum tests. Among all dogs, and in the subset with no corticosteroid administration, CSF TP, CSF TNCC, and CBC N:L ratios were compared across tumor subgroups (primary HS, disseminated HS, meningioma) using Dunn's test of multiple comparisons using rank sums. Overall survival time measured from clinical presentation was estimated using the Kaplan‐Meier product limit method. Dogs alive or lost to follow‐up at the time of data collection were censored in analyses. Rates of definitive versus palliative treatment were compared between primary and disseminated HS cases using Fisher's exact test. Survival estimates were compared according to treatment type (definitive versus palliative) overall and within primary and disseminated HS groups. All tests were 2‐sided, and *P* < .05 was considered statistically significant.

## 3 RESULTS

### 3.1 Signalment

Between 1986 and 2018, 102 dogs were identified with a histopathological diagnosis of CNS HS (Data S1). Tumors consisted of 33 primary brain HS (2 male, 15 castrated male, 16 spayed female; median age, 8.0 years; range, 4.0‐12.0 years), 8 primary spinal cord HS (1 female, 4 castrated male, 3 spayed female; median age, 8.0 years; range, 6.0‐10.0 years), 20 disseminated brain HS (1 male, 2 female, 9 castrated male, 8 spayed female; median age, 8.5 years; range, 4.0‐13.0 years), 25 disseminated spinal cord HS (6 male, 6 castrated male, 13 spayed female; median age, 9.0 years; range, 2.0‐12.0 years), 14 brain HS with no necropsy (1 male, 1 female, 8 castrated male, 4 spayed female; median age, 9.5 years; range, 3.0‐13.0 years), and 2 spinal cord HS with no necropsy (1 castrated male, 3 years age; 1 spayed female, 9 years age). For all CNS HS tumors combined (10 male, 4 female, 43 castrated male, 45 spayed female), the median age was 8.0 years (range, 2.0‐13.0 years). No consistent trend in increasing frequency of CNS HS was seen over the retrospective time period (Data S2); total number of CNS HS (VMTH) cases and their percentage of the total hospital population were 1986‐1996 (14, 0.024%); 1997‐2007 (45, 0.063%); 2008‐2018 (31, 0.034%).

Breed predisposition was determined from 91 cases presented to the UC Davis VMTH with a control hospital population of 222 814 dogs during the study period. Seven breeds had ≥3 CNS HS (Table 1) and represented 57% of all CNS HS cases. Breed predisposition was found in Bernese Mountain dogs, Golden Retrievers, Rottweilers, Shetland Sheepdogs, and Corgis. Corgis and Shetland Sheepdogs had predominantly primary CNS HS, whereas Rottweilers had exclusively disseminated CNS HS. In the uncontrolled cases (Virginia Maryland, n = 11), 3/6 primary CNS HS were in Corgis (2/6) and a Shetland Sheepdog, and 2/5 disseminated CNS HS were in Rottweilers.

**Table 1:** Breed predisposition for CNS HS (breeds with ≥3 CNS HS)

Breed	Hospital %	Histiocytic %	Primary	Dissem	Undet	Exact *P* value	OR (95% CI)
Bernese Mountain Dogs	0.5	8.8	2	6	0	<.001	21.0 (10.1‐43.8)
Corgi	0.7	5.5	3	0	2^a^	.001	8.3 (3.4‐20.5)
Golden Retriever	4.6	14.3	4	6	3^b^	<.001	3.5 (1.9‐6.2)
Labrador	8.8	4.4	2	2	0	.19	0.5 (0.2‐1.3)
Labrador Cross	3.1	3.3	1	1	1^a^	.76	1.1 (0.3‐3.3)
Rottweiler	2.6	15.4	0	14	0	<.001	6.9 (3.9‐12.2)
Shetland Sheepdog	0.9	5.5	3	1	1	.001	6.8 (2.7‐16.7)

Abbreviations: CI, confidence interval; CNS HS, histiocytic sarcoma affecting the central nervous system; Dissem, disseminated; OR, odds ratio; Sig, significance *P* value; Undet, undetermined.

a Normal physical examination, thoracic radiographs and abdominal ultrasound.

b Normal physical examination, thoracic radiographs and abdominal ultrasound (1), multifocal abdominal disease (1), no data (1).

For HS affecting the brain, the majority of tumors were located rostrotentorially. For primary HS there were 19 solitary and 6 multifocal or diffuse rostrotentorial tumors; 2 solitary and 3 multifocal or diffuse infratentorial tumors; 3 tumors affecting both compartments, and 3 of the tumors also had intraventricular involvement. Three tumors also had spinal cord involvement. For disseminated HS affecting brain, there were 9 solitary and 4 multifocal or diffuse rostrotentorial tumors; 3 solitary infratentorial tumors; 3 tumors affecting both compartments; and 2 tumors that involved peripheral cranial nerves (1 with cranial nerves III, IV, V, 1 with cranial nerves III, IV). Two tumors also had spinal cord involvement. Based on MR imaging, involvement of both meninges and brain parenchyma was a consistent finding for intracranial HS and most tumors had broad areas of meningeal contact. All intraventricular tumors were found in animals with multifocal disease, and 1 disseminated tumor involved only cranial nerves III, IV, and V with no parenchymal involvement.

For HS affecting the spinal cord, 7/8 primary HSs were intrinsic compared to disseminated HS where 24/25 tumors were extradural. Vertebral bone involvement was documented in 14/25 of the disseminated tumors with extradural lesions. No apparent regional predisposition was seen with primary HS (2 cervical, 3 thoracic, 2 lumbar, 1 multifocal), but disseminated HS was most common in the thoracic region (3 cervical, 11 thoracic, 6 lumbar, 5 multifocal of which 3 involved thoracic segments). One primary and 2 disseminated tumors also involved brain.

### 3.2 CSF analysis

**Figure 1:**
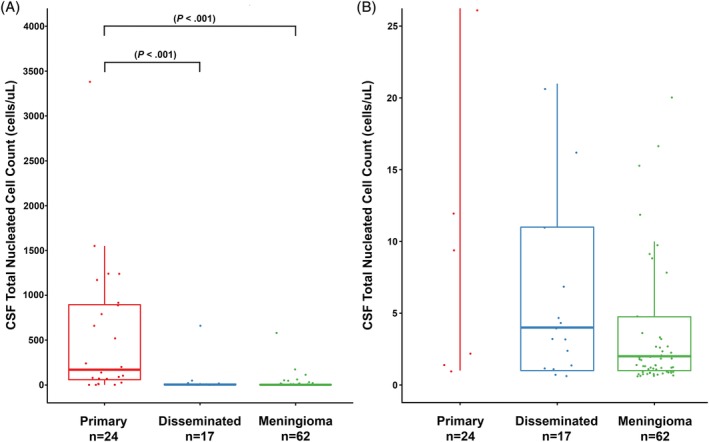
A and B, Box plots of CSF total nucleated cell counts in primary CNS HS, disseminated CNS HS and meningiomas, regardless of corticosteroid administration. B, Expanded TNCC scale to allow assessment of low TNCC data points in disseminated CNS HS and meningioma groups. Boxes correspond to interquartile ranges (25th–75th percentiles); the horizontal line within the box is the median value (50th percentile). Whiskers represent lower and upper adjacent values (i.e. the lowest and highest observations that are still inside the region defined by the following limits: Lower limit: Q1−1.5 × IQR. Upper limit: Q3 + 1.5 × IQR). Significant differences between tumor groups are indicated by brackets. IQR, interquartile range

**Figure 2:**
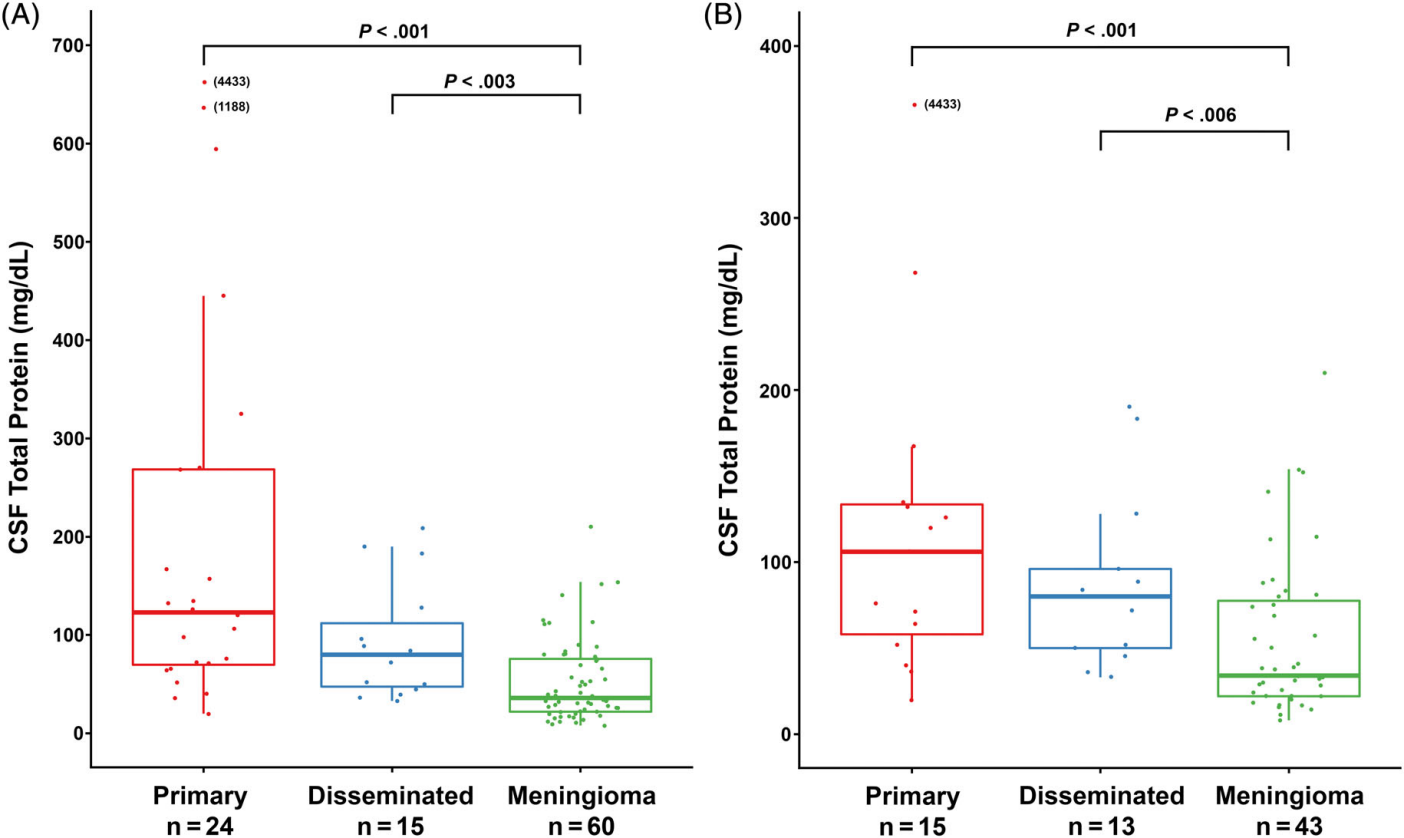
A and B, Box plots of CSF total protein in primary CNS HS, disseminated CNS HS and meningioma. A, All cases regardless of corticosteroid administration. B, Cases with no history of corticosteroid administration. Boxes correspond to interquartile ranges (25th–75th percentiles); the horizontal line within the box is the median value (50th percentile). Whiskers represent lower and upper adjacent values (i.e. the lowest and highest observations that are still inside the region defined by the following limits: Lower limit: Q1−1.5 × IQR. Upper limit: Q3 + 1.5 × IQR). Significant differences between tumor groups are indicated by brackets. Outlying data points are included with values in brackets. IQR, interquartile range

**Figure 3:**
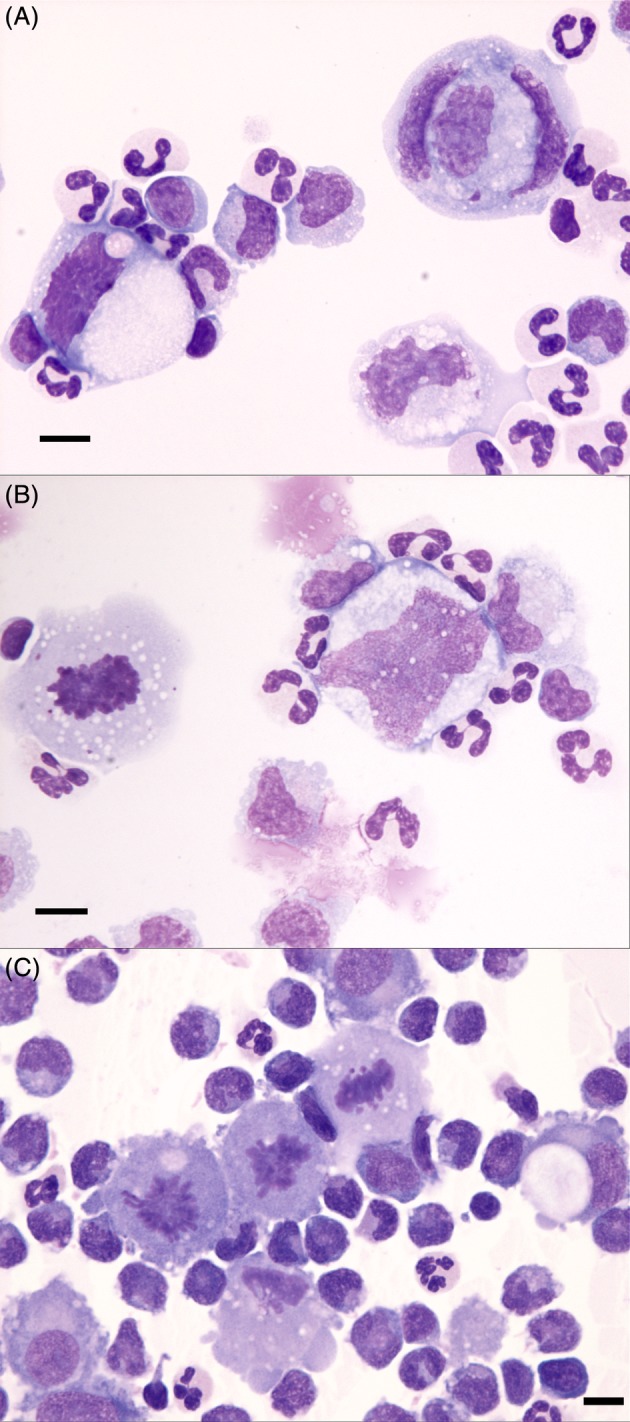
Cerebrospinal fluid cytospin preparations from dogs with primary CNS HS. A, Note the three extremely large, neoplastic, single round cells with large immature and atypical nuclei, admixed with numerous inflammatory cells comprising nondegenerate neutrophils and macrophages. B, There are two large neoplastic histiocytes, one of which is mitotic, admixed with numerous inflammatory cells. C, There is a markedly increased total nucleated cell count (TNCC) and numerous mitoses, which raises the index of suspicion for underlying primary CNS HS and should prompt a search for large, atypical round cells. Wright's‐Giemsa stain, scale bar = 10 μm

Cerebrospinal fluid analysis was available for 24 primary CNS HS (17 brain, 7 spinal cord) and 17 disseminated CNS HS (6 brain, 11 spinal cord) cases (Figures 1, 2, 3; Data S3). Cerebrospinal fluid was collected from the cerebellomedullary cistern in 40/41 brain HS cases with sampling site not noted in 1 case. Cerebrospinal fluid from spinal cord HS cases was collected from the lumbar subarachnoid space in 16/18 and cerebellomedullary cistern in 2/18 cases (Data S3). Total nucleated cell counts were available for all cases, and CSF TP was available for 39/41 samples. Median RBC count for CNS HS was 20 RBC/μL (range, 0‐3900 μL) and 4 RBC/μL (range, 0‐3300 μL) for meningioma samples. Cerebrospinal fluid‐related corticosteroid administration status was reported for all cases, and the rate of corticosteroid administration did not differ significantly (*P* = .58) between HS (12/41, 29.3%) and meningioma (18/62, 29%) cases.

Median CSF TNCC did not differ significantly based on the presence or absence of corticosteroid administration for both HS and meningiomas groups (*P* = .12, *P* = .67, respectively). Median CSF TNCC differed significantly (*P* < .001) across tumor types (Figure 1). Cerebrospinal fluid TNCC was significantly higher in 24 dogs with primary HS (median, 170; interquartile range [IQR], 48‐902) compared to 17 dogs with disseminated HS (median, 4; IQR, 1‐11; *P* < .001) and 62 dogs with meningioma (median, 2; IQR, 1‐5; *P* < .001). No significant difference (*P* = .09) was found between the disseminated HS and meningioma groups.

Median CSF TP differed significantly (*P* = .02) between HS cases that received steroids (209 mg/dL; IQR, 72‐445) and HS cases that did not (82 mg/dL; IQR, 51‐130). Median TP of CSF did not differ significantly (*P* = .75) for meningioma cases based on corticosteroid administration. For all dogs, CSF TP differed significantly (*P* < .001) across tumor types (Figure 2). Median CSF TP was significantly lower in meningioma (median, 36 mg/dL; IQR, 22‐77) compared to primary HS (median, 123 mg/dL; IQR, 69‐269; *P* < .001) and disseminated HS (median, 80 mg/dL; IQR, 45‐128; *P* = .003). No significant difference (*P* = .12) was found between primary and disseminated HS groups. Significance conclusions remained unchanged when CSF TP concentration analysis was restricted to cases with no corticosteroid administration (*P* < .001, *P* = .006, *P* = .34, respectively).

Neoplastic cells were identified in 52% (9/18 primary CNS HS, 4/7 disseminated CNS HS) of cytological preparations for which slides were available for review (Figure 3). Neoplastic cells were identified in 3 samples in which they had not been identified in the initial report. Mitoses were present in 10 samples and only were seen in cases where neoplastic cells also were identified (Figure 3).

### 3.3 Neutrophil to lymphocyte ratio

**Figure 4:**
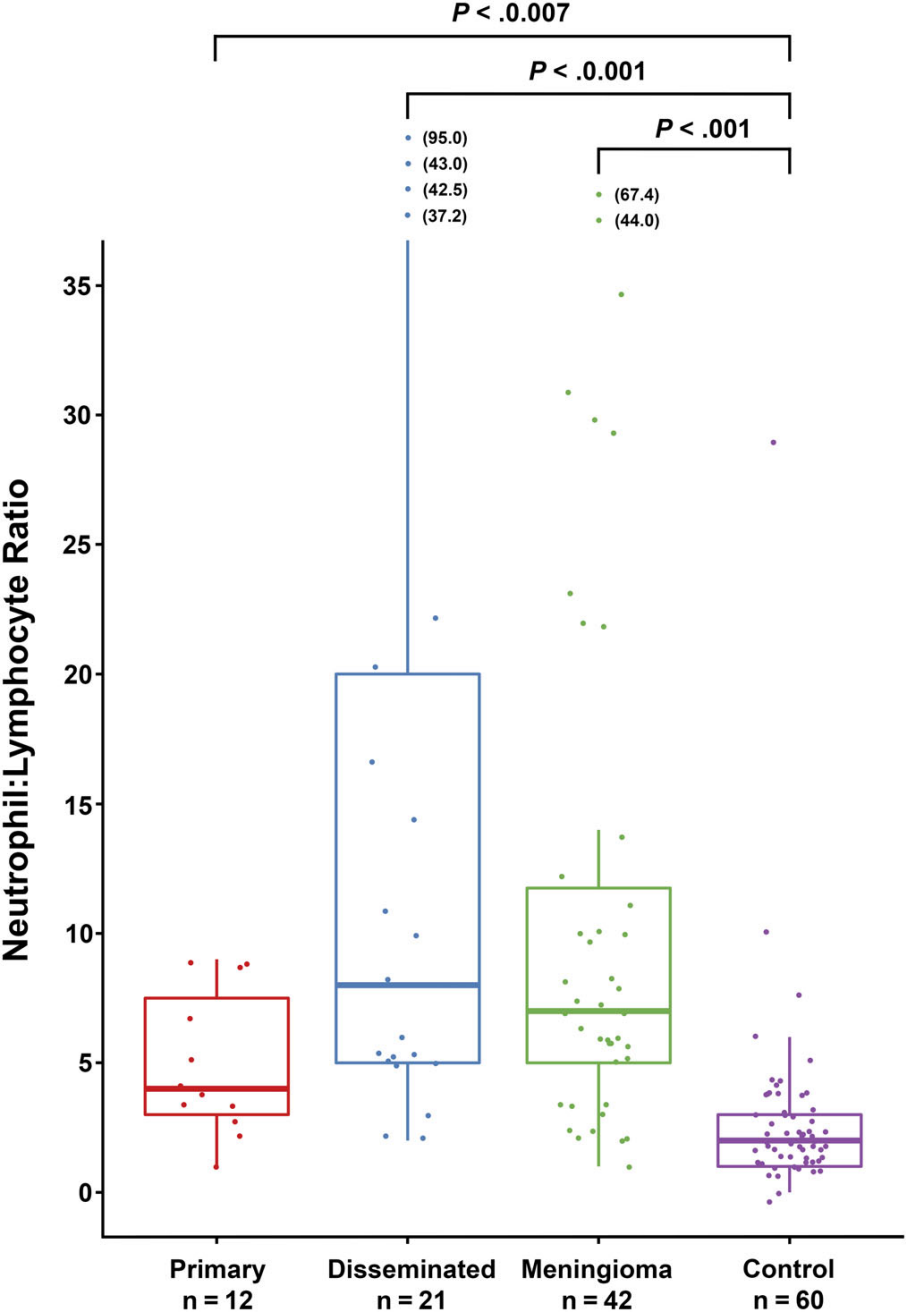
Boxplots of peripheral CBC neutrophil:lymphocyte ratios from cases of primary CNS HS, disseminated CNS HS, meningioma and control (clinically normal) dogs. Whiskers represent +/‐ 1.5 interquartile ranges. Significant differences between tumor groups are indicated by asterisks. Boxes correspond to interquartile ranges (25th–75th percentiles); the horizontal line within the box is the median value (50th percentile). Whiskers represent lower and upper adjacent values (i.e. the lowest and highest observations that are still inside the region defined by the following limits: Lower limit: Q1−1.5 × IQR. Upper limit: Q3 + 1.5 × IQR). Significant differences between tumor groups are indicated by brackets. Outlying data points are included with values in brackets. IQR, interquartile range

Among dogs that did not receive corticosteroids, N:L did not differ significantly (*P* = .08) across tumor types (primary HS, disseminated HS, and meningioma). Median N:L ratio was 4.2 (IQR, 3.7‐8.4) in 12 dogs with primary HS, 8.6 (IQR, 5.5‐20.8) in 21 dogs with disseminated HS, and 7.7 (IQR, 5.4‐12.0) in 42 dogs with meningioma. Median N:L ratio in 60 control dogs (2.6; IQR, 1.8‐2.9) was significantly lower than in the primary HS (*P* = .007), disseminated HS (*P* < .001), and meningioma groups (*P* < .001; Figure 4).

### 3.4 Treatment outcome

**Figure 5:**
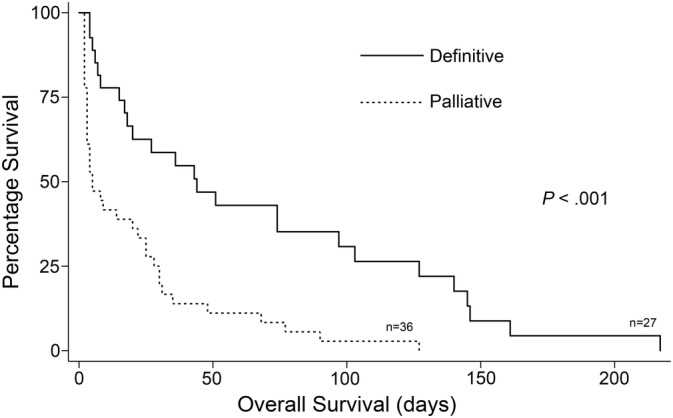
Kaplan‐Meier survival estimates for definitively and palliatively treated histiocytic sarcoma affecting the central nervous system (CNS HS) (primary and disseminated) cases that survived >1 day

Survival time after presentation for neurological signs was available for 96 of 102 cases (33 primary brain, 8 primary spinal cord, 19 disseminated brain, 24 disseminated spinal cord, 10 brain without necropsy, and 2 spinal cord without necropsy). Ninety‐four dogs died and 2 were lost to follow‐up. Treatment modalities and survival data are outlined in Data S1. Median survival time (MST) for all 96 dogs was 4 days (95% CI, 2‐8 days). Thirty‐three dogs (all with palliative or no treatment) survived <1 day and were excluded from further analysis to remove cases without intent to treat. Definitive treatments were done in 27 cases (12 primary HS, 8 disseminated HS, and 7 undefined HS), and palliative treatment in 36 cases (17 primary HS, 15 disseminated HS, and 4 undefined HS). Among dogs surviving >1 day, definitive treatment (MST, 44 days; 95% CI, 17‐97) was associated with significantly (*P* < .001) longer survival compared with palliative treatment (MST, 5 days; 95% CI, 3‐22) (Figure 5). Within this population, significantly longer survival times also were seen when comparing definitively vs palliatively treated primary HS (*P* < .008; MST, 43 days; 95% CI, 5‐127 vs MST, 5 days; 95% CI, 2‐8) and definitively vs palliatively treated disseminated HS (*P* = .03; MST, 27 days; 95% CI, 8‐146 vs MST, 4 days; 95% CI, 2‐14).

## 4 DISCUSSION

Our study defines hospital population‐based breed predispositions for HS affecting the CNS in dogs and defines several clinicopathological characteristics that suggest primary and disseminated HS may have different pathophysiology in the context of CNS disease. Primary and disseminated CNS HS were shown to differ in terms of inflammatory response based on CSF analysis, different breed predisposition, and different anatomical locations of disease, particularly with disease affecting the spinal cord.

The presence of breed predisposition for HS affecting the CNS likely represents underlying genetic susceptibilities, but disease‐related factors may be complex, reflecting not only susceptibility to HS in general but also to development of primary versus disseminated CNS disease. This complexity is apparent in our data in which larger populations of primary and disseminated CNS HS were analyzed. Corgis have been reported to have a high frequency of HS affecting tissues outside the CNS^7^ and also HS affecting the CNS.^13^, ^18^, ^19^ Although limited in numbers, these data and ours suggest that CNS HS in Corgis rarely is associated with disseminated disease, although extra‐CNS disease frequently is reported. This observation is in stark contrast to CNS HS in Rottweilers that accounted for the highest number of HS in our study (16/102, 16%) and where all tumors were associated with disseminated disease outside the CNS as well. Previous reports of CNS HS in Rottweilers also are consistent with disseminated disease,^11^, ^18^, ^26^, ^27^ and this phenotypic variation likely reflects different HS tumor biology in the different groups.

The underlying cell of origin may be different for primary versus disseminated CNS HS. The term histiocyte covers a spectrum of cell types including dendritic antigen presenting cells (APC) or those of macrophage origin.^28^ Most extra‐CNS HS are thought to be of myeloid dendritic APC (interstitial dendritic cell) origin based on their immunophenotype.^1^ The origin of primary CNS HS in dogs is poorly defined, with conflicting reports of expression of both interstitial dendritic cell and macrophage phenotypes.^2^, ^18^, ^19^, ^29^ Definition of dendritic APC and macrophage lineages is complex and controversial and commonly used phenotypic markers (eg, CD1, CD11c, CD11b, CD11d, MHCII) may fail to distinguish dendritic cells from monocytes, macrophages, and even activated microglia, particularly in non‐lymphoid organs.^30^ Interstitial dendritic cells generally are not thought to be present within the brain, but dendritic cells have been reported in the meninges, choroid plexus, and circumventricular organs.^31^ Adding to this complexity, infiltrating inflammatory monocytes or macrophages and subpopulations of resident microglia also may differentiate into dendritic cells within the CNS.^32^, ^33^ Defining the cellular origin of specific CNS histiocytic tumor subgroups will be important for appropriate tumor phenotyping for future genetic studies and for development of appropriately targeted novel therapeutic approaches with respect to what may be 2 distinct tumors.

The previously reported predisposition in Labrador Retrievers was not confirmed in our study, but the suspected predisposition in Shetland Sheepdogs as proposed previously^13^ was confirmed. Miniature Schnauzers have been shown to be predisposed to HS in non‐CNS cases,^6^ and multiple single cases affecting the CNS have been reported.^14^, ^18^, ^19^, ^34^, ^35^ Only 2 Miniature Schnauzers were reported in our series, both with disseminated disease, with no defined breed predisposition based on this limited number of cases.

Primary CNS HS was associated with an inflammatory phenotype compared to disseminated CNS HS (and meningioma) based on CSF TNCC. We can speculate that the specific cell lineage of primary versus disseminated disease is associated with a more pro‐inflammatory phenotype, but other factors may be associated with anatomical localization of the neoplastic process. Primary HS spinal cord tumors were predominantly intrinsic compared to extradural disseminated tumors, and a logical argument can be made that these primary tumors would be more likely to stimulate an inflammatory response, reflected in CSF pleocytosis within the subarachnoid space. However, other intrinsic and intradural spinal cord tumors generally are not associated with the marked pleocytosis seen with primary CNS HS,^36^, ^37^ making anatomical location unlikely to be the only factor. In the absence of a defined intracranial subdural space, both primary and disseminated HS involve both the meninges and the brain parenchyma, and most other intracranial neoplasia is not associated with marked CSF pleocytosis,^22^, ^23^, ^24^ also supporting an argument for inherent differences in the pro‐inflammatory nature of the primary versus disseminated HS subtypes. Articular or periarticular HS also has been associated with a marked inflammatory response,^8^ and associations have been made between prior injury and inflammation involving the joint and the development of HS.^38^, ^39^ No direct evidence supports underlying inflammation as a predisposing factor for the development of HS, but the link between inflammation and a variety of cancers is well documented.^40^ Further investigation is warranted to determine whether preexisting inflammation may be a precursor for primary CNS HS and whether specific breeds may have genetic predispositions consistent with this pathogenic mechanism.

Systemic inflammatory responses associated with cancer are well documented, and increases in peripheral blood N:L ratios have been used widely as an indicator of tumor‐associated inflammation.^41^ Increased N:L ratios have been associated with poorer overall survival in a variety of cancers in humans,^42^ including intracranial gliomas.^43^ The N:L ratios were significantly increased in all tumor subgroups in our study compared to normal reference ranges, but the marked pro‐inflammatory phenotype of primary versus disseminated CNS HS reflected in the CSF TNCC was not apparent in peripheral blood N:L ratio findings. Although no significant differences were found among the relatively low number of samples in the different tumor groups, the disseminated HS and meningioma N:L ratios (median, 8.6 and 7.7, respectively) were higher than in the primary HS group (median, 4.2). Specific underlying mechanisms driving cancer‐derived systemic inflammation are poorly understood, and the anatomical location of the primary HS tumors or lack of systemically acting, tumor‐related factors may contribute to a lesser systemic response compared to that of the disseminated HS and meningiomas. The presence of neoplastic cells in CSF did not appear to be related to the severity of CSF pleocytosis, and neoplastic cells were identified in both primary and disseminated HS CSF samples. Although mitoses may be seen in non‐neoplastic CSF‐nucleated cells, they are uncommon, and the strong association of mitoses with the presence of neoplastic cells in HS is notable. Utility of CSF as a diagnostic tool for intracranial mass lesions is not always appreciated, but the markedly high incidence (>50%) of diagnostic atypical cells, and the significant difference in CSF TNCCs between primary HS and meningiomas underscores the value of CSF analysis when imaging‐based data are suggestive of these tumor types as primary differential diagnoses. Interestingly, all 3 CSF samples containing tumor cells that were not identified on initial examination had both markedly increased TNCC and scattered mitoses, which, in the appropriate clinical context, should increase the index of suspicion for primary CNS HS and the presence of neoplastic cells, respectively. The presence of eosinophils was a common finding in CNS HS CSF (35%), but this finding was not specific for an HS subtype and also was seen in CSF samples of meningioma cases, as reported previously.^23^ Corticosteroid usage and dosage were variable among cases, but this difference likely did not detract from overall conclusions related to CSF pleocytosis and survival data. Counterintuitively, HS cases that received corticosteroids had significantly higher CSF TP, and although not significant, CSF TNCC also was higher in the steroid administration HS group. This finding may be reflective of a higher number of primary (and more likely inflammatory) cases (8/41 versus 3/41 disseminated HS cases) receiving corticosteroids, possibly associated with more severe clinical signs.

Limited data are available regarding treatment and survival for dogs with CNS HS, but anecdotal reports suggest prognosis is generally poor. Case series of CNS HS with >3 dogs have reported MST of 3 days (n = 19),^13^ 14 days (n = 4),^14^ and 30 days (n = 5)^15^ with no distinction between definitive and palliative treatment. Low sample numbers and marked variability in tumor locations and treatment modalities preclude any detailed analysis of therapeutic outcome in our study, but general comments can be made. Median survival for all dogs for which data were available (n = 96) was similar to previous reports at 4 days, but 30 dogs had survival times of 1 day. Removing these (presumably euthanized at diagnosis) cases resulted in median survivals of 44 days for definitively (surgery, radiation therapy, lomustine chemotherapy) treated cases (n = 27) and 5 days for palliative or no treatment cases (n = 36). “Dedicated owner” effects are likely to have affected the definitively treated survival outcomes, but the maximum documented survival time still was <8 months in a dog with a primary intracranial HS treated with surgery and lomustine, and only 8 of 96 dogs had survival times >4 months. Primary CNS HS is an extremely rare tumor in humans and carries a similarly poor prognosis with survival after definitive therapy typically <1 year.^44^, ^45^ Limited data are available to define the incidence of primary versus secondary CNS HS in humans, but most reports involve primary tumors. Interestingly, a marked inflammatory infiltrate also has been described for these primary tumors in humans similar to their counterparts in dogs,^46^, ^47^, ^48^, ^49^ suggesting that they may have some common underlying characteristics.

Our study conclusions are limited by relatively small sample sizes in tumor and treatment subgroups, variability in defined survival end points and potential for inappropriate categorization of primary tumors, even with comprehensive necropsy examination. However, the significant differences defined between primary and disseminated CNS HS particularly in terms of breed predisposition and inflammatory response within the CNS are notable. Advances in treatment of a variety of cancers in humans by manipulation of inflammatory and associated immune‐cell modulation have resulted in new optimism about several previously intractable cancers. Primary CNS HS in dogs appears to carry a poor prognosis regardless of standard therapeutic interventions. Further evaluation of the inflammatory environment present in these tumors may provide critical insight into the biology of CNS HS in dogs and the potential for the development of novel therapeutic approaches.

### Conflict of Interest Declaration

Authors declare no conflict of interest.

### Off‐label Antimicrobial Declaration

Authors declare no off‐label use of antimicrobials.

### Institutional Animal Care and Use Committee (IACUC) or Other Approval Declaration

Authors declare no IACUC or other approval was needed.

### Human Ethics Approval Declaration

Authors declare human ethics approval was not needed for this study.

## Supporting information


**Appendix S1**: Supplementary Material

**Appendix S2**: Supplementary Material

**Appendix S3**: Supplementary Material
