# Supplementary material for: Clinicopathological characteristics of histiocytic sarcoma affecting the central nervous system in dogs
Source: J Vet Intern Med. 2020 Jan 10;34(2):828–37. doi: 10.1111/jvim.15673 (PMC7096655; doi:10.1111/jvim.15673)
Supplement: Supplementary file 1 — Appendix S1: Supplementary Material [file JVIM-34-828-s001.pdf]

Primary Brain	Breed	Sex	Age (years)	Definitive Tx	Survival (days)
1	Labrador Retriever	MC	9	Rad	44
2	Pembroke Welsh Corgi	MC	5	P	1
3	English Pointer	M	9	P	3
4	Beagle	MC	4	Sx	43
5	Bernese Mountain dog	M	6	P	3
6	Golden Retriever	FS	9	Sx	5
7	Golden Retriever Mix	FS	9	Sx	127
8	Golden Retriever	FS	6	P	1
9	Golden Retriever	FS	7	P	2
10	Tibettan Terr	MC	6	P	28
11	Shetland Sheepdog	MC	11	P	3
12	Shetland Sheepdog	MC	6	Sx, Rad, CCNU	145
13	Standard Poodle	FS	7	P	4
14	Pembroke Welsh Corgi	MC	11	P	1
15	Labrador Retriever MixX	FS	5	P	20
16	French Bulldog	MC	6	Sx	18
17	Scottish Terrier	FS	9	P	1
18	Staffordshire Terrier	FS	7	P	1
19	Dalmation X Pitbull	FS	8	P	4
20	Pit Bull Mix	FS	6	P	2
21	Bernese Mountain dog	FS	8	None	1
22	Maltese	FS	9	P	30
23	Smooth Collie	FS	11	None	1
24	CardiganWelsh Corgi	MC	6	Sx, CCNU	97
25	Italian Greyhound	FS	7	P	1
26	Golden Retriever	MC	8	Rad, CCNU	140
27	TerrierMix	MC	7	P	25
28	Pomeranian Mix	FS	9	Rad	4
29	Pembroke Welsh Corgi	MC	9	P	31

Definitive Tx	Number	Median Survival (days)
Surgery +/-	16	58.5
Radiation therapy+/-	7	74
CCNU Chemotherapy+	11	97

30	Shetland Sheepdog	MC	10	P	1
31	Mix	FS	9	P	9
32	Beagle	MC	12	Sx, Rad, CCNU	74
33	English Setter	MC	8	CCNU	6

Primary SC

1	Shetland Sheepdog	MC	6	P	48
2	Labrador Retriever	FS	10	P	5
3	Kuvasz	MC	10	Sx	15
4	Briard	F	8	P	1
5	Greyhound	MC	6	P	3
6	German Shepherd dog	FS	8	None	1
7	Poodle X Maltese	MC	7	None	1
8	Pembroke Welsh Corgi	FS	8	P	77

Disseminated Brain

1	Siberian Husky	FS	13	Rad	17
2	Golden Retriever	M	10	None	1
3	Rottweiler	F	4	P	14
4	Miniature Schnauzer	MC	13	P	3
5	Rottweiler	FS	10	None	2
6	Bernese Mountain dog	FS	9	NA	NA
7	Rottweiler	MC	5	None	1
8	Great Pyrenees	FS	9	P	35
9	Rottweiler Mix	MC	12	CCNU	27
10	Rottweiler	MC	6	P	1
11	Labrador Retriever Mix	MC	6	P	22
12	Bernese Mountain dog	FS	8	CCNU	146
13	English Setter	FS	7	P	1
14	Rottweiler	MC	10	CCNU	161

15	Shetland Sheepdog	MC	10	None	1
16	Bernese Mountain dog	FS	7	None	127
17	Labrador Retriever	MC	10	None	1
18	Rottweiler	MC	6	P	1
19	Rottweiler	F	8	P	1
20	Bernese Mountain Dog	FS	8	P	4

Disseminated SC

1	Flat Coated Retriever	MC	11	P	1
2	Rottweiler	M	8	P	1
3	Golden Retriever	FS	9	P	1
4	Bernese Mountain dog	FS	2	NA	NA
5	Rottweiler	M	6	P	1
6	Rottweiler	M	7	P	5
7	Rottweiler	FS	8	Sx	8
8	Golden Retriever	FS	10	None	1
9	Rottweiler	FS	8	Sx	36
10	Rottweiler	FS	6	None	2
11	Labrador Retriever	FS	3	P	1
12	Golden Retriever	FS	12	P	2
13	Great Pyrenees	MC	9	P	2
14	Leonberger	FS	12	P	1
15	Rottweiler	FS	10	P	1
16	Rottweiler	M	8	P	1
17	Golden Retriever	M	10	P	1
18	Great Dane	FS	6	P	1
19	Rottweiler	MC	5	P	2
20	Bernese Mountain dog	MC	9	None	2
21	Bernese Mountain dog	M	5	P	1
22	Miniature Schnauzer	FS	9	P	1
23	Golden Retriever	MC	9	P	68

24	Labrador Retriever	MC	10	Sx, CCNU	8
25	Mix	FS	11	CCNU	20

Brain No Necropsy

1	Labrador Retriever Mix	MC	8	Sx	NA
2	Standard Poodle	MC	6	Sx	NA
3	Golden Retriever	MC	12	Sx	120
4	Shih Tzu	MC	10	Sx, CCNU	217
5	Pembroke Welsh Corgi	FS	13	Sx	51
6	Pembroke Welsh Corgi	FS	9	P	3
7	Golden Retriever	MC	10	Sx	74
8	Cavalier King Charles	FS	6	Sx, Rad	103
9	American Eskimo	MC	10	P	NA
10	Englidh Springer Spaniel	MC	10	NA	NA
11	Mix	M	13	NA	90
12	Shetland Sheepdog	FS	3	NA	30
13	Skye Terrier	F	5	NA	NA
14	American Pit Bull	MC	4	NA	NA

SC No Necropsy

1	Golden Retriever	FS	9	None	25
2	German Shepherd dog	MC	3	Sx	4

CCNU= CCNU chemotherapy
NA=Data not available
P= Palliative
Rad= Radiation therapy
S= Symptomatic
Survival= Survival from time of presentation
Sx= Surgical resection
