# Supplementary material for: Clinicopathological characteristics of histiocytic sarcoma affecting the central nervous system in dogs
Source: J Vet Intern Med. 2020 Jan 10;34(2):828–37. doi: 10.1111/jvim.15673 (PMC7096655; doi:10.1111/jvim.15673)
Supplement: Supplementary file 2 — Appendix S2: Supplementary Material [file JVIM-34-828-s002.pdf]

Temporal frequency of CNS Histiocytic sarcoma (VMTH UC Davis)

Total Hospital Population		Primary Br	Primary SC	Disseminated Br	Disseminated SC	Unknown	Total	% Total Population
59,327	**86-96**	2	0	4	8	0	**14**	0.024
71,079	**97-07**	12	5	9	10	9	**45**	0.063
92408	**08-18**	14	2	4	5	6	**31**	0.034

Br = Brain
SC = Spinal cord

Predisposed breed frequency

Corgi	Shetland Sheep	BMD	Rottweiler	G Retriever
**1** (311)[0.3%]	**0** (839) [0%]	**1** (192) [0.5%]	**5** (2454) [0.2%]	**3** (3164) [0.1%]
**1** (452) [0.2%]	**4** (697) [0.6%]	**3** (409) [0.7%]	**7** (2203) [0.3%]	**7** (3760) [0.2%]
**3** (783) [0.4%]	**1** (387) [0.3%]	**4** (418) [1.0%]	**2** (1148) [0.2%]	**3** (3398) [0.1%]

Numbers = No of breed animals with HS
(x) = Total number of breed animals in time period
[x] = % HS for breed in time period
