# Supplementary material for: Clinicopathological characteristics of histiocytic sarcoma affecting the central nervous system in dogs
Source: J Vet Intern Med. 2020 Jan 10;34(2):828–37. doi: 10.1111/jvim.15673 (PMC7096655; doi:10.1111/jvim.15673)
Supplement: Supplementary file 3 — Appendix S3: Supplementary Material [file JVIM-34-828-s003.pdf]

CNS Histiocytic Sarcoma

Case No	Site	CSF protein mg/dL	RBC /uL	TNCC /uL	Neut %	Small mono %	Large mono %	Eosinophil	Corticosteroids	Mitoses	Neoplastic Cells
**Primary Brain**											
1	C	40	10	79	13	47	7	37 eos	N		NA
4	C	270	140	1170	80	14	6		Y		NA
7	C	71	1	1	3	47	47	6 eos	N		N
10	C	106	2	888	52	41	7		N		NA
11	C	120	320	139	11	31	57	1 eos	N	Y	Y
14	C	135	220	1550	19	1	80	rare eos	N	Y	Y
15	C	76	13	240	8	6	77	9 eos	N		Y
17	C	594	373	3380	56	5	39		Y	Y	Y
19	C	36	3	9	30	13	56	1% eos	N		N
22	C	132	0	12	0	78	22		N		N
26	C	20	2	1	0	rare	0		N		N
27	C	157	1	201	36	12	52		Y	Y	Y
29	C	126	0	71	10	43	47		N		N
30	C	52	28	520	7	17	76		N	Y	Y
31	C	98	25	103	64	20	16		Y		NA
32	C	268	6	1,239	35	14	45	16 eos	N		N
33	C	64	0	916	80	rare	12	7 eos	N		N
**Primary SC**											
1	L	445	56	69	rare				Y		NA
2	L	167	340	90	6	89	5		N		Y
3	L	66	600	2	NA	NA	NA		Y		NA
4	L	1188	3900	790	68	22	10		Y		N
5	C	72	3	660	15	12	68	5% eos	Y	Y	Y
6	L	4,433	2,330	1,240	25	30	21	24% eosin	N	Y	Y
8	L	325	368	26	13	9	68	5 eos	Y		N

Diss Brain

1	NA	1.3349 RI	1	3	3	20	77		Y		NA
3	C	45	1	3	8	47	25	20 eos	N		NA
8	C	36	152	1	13	47	40		N		N
18	C	NA	27	11	3	90	7		N		N
19	C	39	40	21	0	62	16		Y	Y	Y
20	C	52	3	16	0	46	54		N		N

DIss SC											
1	C	72	3	660	15	12	68	5% eos	N		NA
5	L	190	20	4	10	33	57		N		NA
7	L	96	0	4	5	87	8		N		NA
8	L	183	1710	5	80	11	9		N		NA
9	L	33	6	1	0	56	44		N		NA
15	L	50	17	1	4	42	54		N		NA
17	L	128	1	2	?	?	?		N		NA
18	L	80	31	1	1	92	7		N		NA
23	L	89	610	7	0	45	55		N		Y
24	L	209	553	49	5	43	52		Y	Y	Y
25	L	84	1,860	1	rare	8	88	4 eos	N	Y	Y

Meningioma

Case No		CSF protein mg/dL	RBC /uL	TNCC /uL	Neut %	Small mono %	Large mono %	Eosinophil	Corticosteroids
**Meningioma IC**									
1	C	8	1	1	0	0	100		N
2	C	11	1	1	0	15	85		N
3	C	17	1	1	0	72	28		N
4	C	16	80	1	0	0	100		N
5	C	20	1	2	0	91	9		N
6	C	22	0	1	0	68	32		N
7	C	22	468	1	NA				N

8	C	18	1	1	0	83	17		N
9	C	9	1	1	0	1 counted	0		Y
10	C	26	89	1	NA	NA	NA		N
11	C	115	10	2	0	27	73		N
12	C	75	113	1	NA	NA	NA		N
13	C	88	13	2	0	54	46		N
14	C	32	730	1	0	rare	NA		N
15	C	30	7	2	0	2	98		N
16	C	34	1	1	0	rare	few		N
17	C	31	1	1	0	56	44		Y
18	C	43	2	1	0	33	67		Y
19	C	22	1	1	18	36	46		N
20	C	24	1	1	9	44	47		N
21	C	20	1	1	18	69	13		N
22	C	17	20	1	20	40	40		N
23	C	15	0	1	4	76	20		Y
24	C	12	1	1	3	42	55		Y
25	C	12	1	1	5	45	50		Y
26	C	50	0	3	4	45	51		N
27	C	113	6	2	5	30	65		N
28	C	33	68	1	34	40	26		N
29	C	55	0	1	6	34	60		N
30	C	90	0	2	12	50	37	1 eos	N
31	C	41	1	1	2	7	91		N
32	C	39	1	3	2	62	30	6 eos	N
33	C	29	4	2	1	40	58	1 eos	N
34	C	31	1	1	1	0	99		N
35	C	27	1	1	3	34	63		Y
36	C	210	0	4	2	28	70		N
37	C	57	9	2	7	35	58		N
38	C	81	1	2	10	61	29		N
39	C	26	3,130	3	70	19	9	2 eos	Y

40	C	112	2	1	1	15	84		Y
41	C	33	4	1	4	71	25		Y
42	C	74	3300	9	72	13	15		N
43	C	38	1	5	53	27	20		N
44	C	34	420	31	72	12	15	1 eos	N
45	C	154	1170	580	88	1	11		N
46	C	69	750	15	75	2	23		N
47	C	80	4	9	4	81	15		N
48	C	28	1	61	1	77	16	7 eos	N
49	C	152	277	12	35	14	51		N
50	C	ND	9	20	22	34	44		N
51	C	80	1	8	15	5	80		Y
52	C	NA	420	17	78	4	18		N
53	C	53	114	52	74	0	26		Y
54	C	111	22	114	85	7	8		Y
55	C	48	2	10	74	0	26		Y
56	C	78	358	173	87	0	12	1 eos	Y
**Meningioma SC**									
57	L	83	102	3	1	80	19		N
58	L	141	39	47	2	94	4		N
59	C	14	171	1	21	42	37		N
60	L	38	64	1	0	79	21		N
61	L	66	600	2	NA	NA	NA		Y
62	L	52	885	3	5	20	75		Y

NA = Data not available
N = No
Y = Yes
SC = Spinal cord
IC = Intracranial
RI = Refractive index
